# Rifoligotyping assay: an alternative method for rapid detection of rifampicin resistance in *Mycobacterium tuberculosis* isolates from Morocco

**DOI:** 10.1080/13102818.2014.975569

**Published:** 2014-11-11

**Authors:** Imane Chaoui, Naima Atalhi, Radia Sabouni, Mohammed Akrim, Mohammed Abid, Saaid Amzazi, Mohammed ElMzibri

**Affiliations:** ^a^ Unit of Biology and Medical Research, National Center of Energy, Sciences and Nuclear Techniques, Rabat, Morocco; ^b^ Laboratory of Biochimestry and Immunology, Faculty of Sciences, Mohammed V University, Rabat, Morocco; ^c^ National Tuberculosis Reference Laboratory, National Institute of Hygiene, Rabat, Morocco; ^d^ Laboratory of Mycobacteria Genetics, Research Service, Pasteur Institute, Tanger, Morocco

**Keywords:** Morocco, MTB, rifampicin resistance, reverse line blot assay, multi drug resistant

## Abstract

One of the greatest threats to global tuberculosis (TB) control is the growing prevalence of drug resistant strains. In the past decades, considerable efforts have been made upon the development of new molecular technologies and methodologies for detection of drug resistance in *Mycobacterium tuberculosis* (MTB). A sensitive, specific reverse line blot assay, called rifoligotyping (RIFO), for the detection of genotypic resistance to rifampicin (RIF), was designed and evaluated. RIFO includes oligonucleotide probes specific for wild-type and mutant sequences, allowing specific and sensitive detection of both genotypes in a single assay. The RIFO was applied on 500 MTB isolates from Morocco. The results of the RIFO showed a good sensitivity (90.9%) and high specificity (100%); the positive and negative predictive values were 100% and 96.1%, respectively. This rapid, simple, economical assay provides a practical alternative for RIF genotyping, especially in low-income countries, to improve TB control and management.

## Abbreviations

*Bp*: Base pair
*DR-TB:* Drug-resistant tuberculosis
*DST:* Drug susceptibility testing
*MDR-TB:* Multidrug-resistant tuberculosis
*MTB:* *Mycobacterium tuberculosis*
*PCR:* Polymerase chain reaction
*RIF:* Rifampicin
*RIFO:* Rifoligotyping
*RIF^R^:* Rifampicin resistant
*RRDR:* Rifampicin resistance determining region
*Rt:* Room temperature
*TB:* Tuberculosis
*WHO:* World Health Organization
*XDR:* Extremely drug resistant

## Introduction

Tuberculosis (TB) is a treatable airborne infectious disease with almost 500,000 multidrug resistant tuberculosis (MDR-TB) cases emerging annually, of which 5%–7% become extremely drug resistant (XDR).[1,2] Thus, rapid diagnosis of drug resistant tuberculosis (DR-TB) is one of the cornerstones for global TB control, as it allows adequate and efficient therapeutic interventions.[3,4] Basically, the slow growth of the tubercle bacillus is the greatest obstacle to rapid diagnosis of the disease.[4] Usually, the gold-standard of TB diagnosis by culture takes weeks to become positive and even with the up-to-date automated fluid culture methods, it takes an average of 14 days.[5–7] Another 14 days are required for additional testing to get the information on drug susceptibility.[7–9]

In the past decades, major advances in molecular biology tools and the availability of new information generated after deciphering the complete genome sequence of *Mycobacterium tuberculosis* (MTB) increased our knowledge of the mechanisms of resistance to the main anti-TB drugs and showed that specific genetic mutations were associated with drug resistance.[10–12]

Rifampicin (RIF), discovered in 1963, is the most powerful bactericidal drug against TB, the most potent sterilizing drug available and a key component for TB treatment.[11–13] RIF resistance (RIF^R^) is particularly amenable to rapid molecular detection, since more than 95% of all RIF^R^ strains contain mutations localized within the 81 bp core region of the bacterial RNA polymerase *rpoB* gene, known as an RIF^R^ determining region (RRDR), which encodes the active site of the enzyme.[11,12,14,15] Moreover, mutations that occur in this region are highly predictive of RIF^R^, whereas susceptible isolates almost have the same wild-type nucleotide sequence.[11,12,16–18] Interestingly, RIF^R^ is strongly, although not invariably, a surrogate marker for MDR-TB (defined by concomitant resistance to isoniazid – another key anti-TB agent).[12,19] Recently, there has been considerable progress in the development of novel diagnostic tools, especially molecular methods, for direct detection of MTB in clinical specimens.[4,20,21] These methods based on nucleic acid amplification (NAA) of different targets, aim to identify the MTB complex and detect specific genetic mutations that are most frequently associated with phenotypic resistance to one or more drugs.[22–24] In general, these molecular methods available as commercials kits are recommended since they have a better level of standardization, reproducibility and automation. However, some aspects, such as cost-efficiency and the appropriate setting for the implementation of these techniques, are not yet well established. The World Health Organization (WHO) strongly supports the implementation and universal use of these new molecular methods, especially GenXpert MTB/RIF, to detect resistance to RIF and thus predict MDR-TB.[25] GenXpert MTB/RIF, an automated molecular test for MTB detection and resistance to RIF, uses heminested real-time polymerase chain reaction (PCR) assay to amplify an MTB-specific sequence of the *rpoB* gene, which is probed with molecular beacons for mutations within the RRDR.[26–28]

Alternatively, a home NAA method called rifoligotyping (RIFO) provides a practical alternative to sequencing and to GenXpert MTB/RIF, especially in low-income countries. It is designed to detect genotypic resistance within the *rpoB* core region in RIF resistant strains.[29] This approach is based on the principle of reverse hybridization and simultaneously detects a wide range of mutations affecting six independent codons of the *rpoB* gene. The RIFO includes oligonucleotide probes specific for both wild-type and mutant sequences, allowing sensitive detection of all genetic mutations in a single assay.

This study was planned to optimize the RIFO in our setting, using DNA samples with known sequences, and then to apply it on a collection of MTB strains from Morocco. The aim was to determine the sensitivity and specificity of the RIFO assay for accurate and rapid detection of RIF^R^ in smear-positive pulmonary clinical specimens, to predict MDR strains and improve the control and management of TB.

## Materials and methods

### Mycobacterial strains

A collection of 500 isolates from patients with pulmonary TB from different cities in Morocco were collected from the National Institute of Hygiene and Institute Pasteur and used for optimization and validation of the RIFO. The MTB strain H37Rv was used as a reference strain for wild-type genotype. Well-defined DNA samples (from laboratory collection) with diverse genetic mutations were used as mutant controls.

### Drug susceptibility testing

The drug susceptibility testing (DST) of all isolates was determined by the conventional Löwenstein–Jensen medium proportion method described by Canetti et al.[30,31] The concentration of RIF in the medium was 40 μg/mL.

### Template DNA isolation

DNA templates for genotyping were prepared from scraped colonies suspended in 400 μL of 1x TE buffer (10 mmol/L Tris-HCl pH: 8.0, 1 mmol/L ethylenediaminetetraacetic acid (EDTA) pH 8.0, followed by heat inactivation at 100 ºC for 10 min, and stored at –20 °C until further use.

### PCR amplification

The hot Spot region of the *rpoB* gene was amplified by PCR using the primers rpoB-For (5′-Biotin-TGGTCCGCTTGCACGAGGGTCAGA-3′) and rpoB-Rev (5′-Biotin-CTCAGGGGTTTCGATCGGGCACAT-3′). For the PCR reaction, 50 μL of the following mixture was used: PCR buffer 10X (Invitrogen, Saint Aubin, France), 2 mmol/L of MgCl_2_, 2.5 mmol/L of each deoxynucleoside triphosphate (dNTP), 10 μmol/L of each of the biotinylated primers: rpoB-For and rpoB-rev, 1 U/μL of Taq DNA polymerase (Invitrogen, Saint Aubin, France) and 10–100 ng of DNA (2 μL of lysate). The PCR was run for 30 cycles of 96 °C for 1 min, 62 °C for 1 min, 72 °C for 1 min followed by final extension at 72 °C for 10 min. Amplicon was used for RIFO only if a single band of 465 bp was clearly visible.

### Rifoligotyping assay

#### Blotting of probes

The amino-linked oligonucleotide probes listed in Table 1 were covalently bound to a Biodyne C membrane (Pall Corporation) by a previously described methodology.[32] Briefly, the membrane was activated by incubation with 16% EDAC (1-ethyl-3-(3-dimethylaminopropyl)carbodiimide, Calbiochem) for 10 min. The oligonucleotides were diluted to 200 nmol/L in 0.5 M NaHCO_3_ and applied to the membrane in parallel lines, using a miniblotter MN45 (Immunetics, USA). After 1 min of incubation at room temperature, the membrane was inactivated with 100 mmol/L NaOH for 10 min and washed in 2x saline-sodium phosphate-EDTA (SSPE) 0.1% sodium dodecyl Sulfate (SDS) for 5 min at 60 °C. Then, the membrane was sealed in plastic bags containing 20 mmol/L EDTA (pH 8) for further use.

**Table 1.** Sequences of oligonucleotide probes used in the RIFO assay.

Lines	Gene probe	Sequences
1	509–514 wt	5′- NH_3_^+^-AGC CAG **CTG** AGC CAA TTC AT-3′
2	514–520 wt	5′- NH_3_^+^-TTC ATG **GAC** CAG AAC AAC CCG -3′
3	521–525 wt	5′- NH_3_^+^-GCT **GTG** GGG TTG ACC -3′
4	524–529 wt	5′- NH_3_^+^-TTG ACC **CAC** AAG CGC CGA-3′
5	530–534 wt	5′- NH_3_^+^-CTG **TCG** GCG CTG GGG C-3′
6	531 TTG	5′- NH_3_^+^-CTG **TTG** GCG CTG GGG C-3′
7	531 TGG	5′- NH_3_^+^-CTG **TGG** GCG CTG GGG C-3′
8	533 CCG	5′- NH_3_^+^-GCG CCG GGG **CCC** G-3′
9	526 TAC	5′- NH_3_^+^-TTG ACC **TAC** AAG CGC CGA-3′
10	526 GAC	5′- NH_3_^+^-TTG ACC **GAC** AAG CGC CGA-3′
11	526 CGC	5′- NH_3_^+^-TTG ACC **CGC** AAG CGC CGA-3′
12	526 CTC	5′- NH_3_^+^-TTG ACC **CTC** AAG CGC CGA-3′
13	526 TGC	5′- NH_3_^+^-TTG ACC **TGC** AAG CGC CGA-3′
14	526 CCC	5′- NH_3_^+^-TTG ACC **CCC** AAG CGC CGA-3′
15	526 AAC	5′- NH_3_^+^-TTG ACC**AAC** AAG CGC CGA-3′
16	526 ACC	5′- NH_3_^+^-TTG ACC **ACC**AAG CGC CGA-3′
17	526 CAG	5′- NH_3_^+^-TTG ACC **CAG** AAG CGC CGA-3′
18	511 CCG	5′- NH_3_^+^-AGC CAG **CCG** AGC CAA TTC AT-3′
19	511 CGG	5′- NH_3_^+^-AGC CAG **CGG** AGC CAA TTC AT-3′
20	513 CTA	5′- NH_3_^+^-AGC CAG CTG AGC **CTA** TTC AT-3′
21	513 CCA	5′- NH_3_^+^- AGC CAG CTG AGC **CCA** TTC AT-3′
22	514 ITTC	5′- NH_3_^+^-CTG AGC CAA TTC **TTC** ATG GAC-3′
23	516 GTC	5′- NH_3_^+^-TTC ATG **GTC** CAG AAC AAC CCG-3′
24	516 TAC	5′- NH_3_^+^-TTC ATG **TAC** CAG AAC AAC CCG-3′
25	Δ516−517	5′- NH_3_^+^-CAA TTC ATG AAC AAC CCG C-3′
26	518ΔAAC	5′- NH_3_^+^-CAG AAC CCG CTG TCG G-3′
27	522 TTG	5′- NH_3_^+^-G CTG **TTG** GGG TTG ACC-3′

Note: wt – wild type.

#### Hybridization

A volume of 10 μL of PCR product was diluted in 150 μL of 2× SSPE 1%SDS buffer, denatured at 99 °C for 10 min and cooled on ice. The heat-denatured single-stranded PCR products were applied on the membrane mounted in the mini blotter. Of the 45 slots in the apparatus, two were reserved, respectively, for positive (strain H37Rv pan-susceptible) and negative (water) controls. The first and the last slots were filled with 160 μL of 2× SSPE 1%SDS buffer. The remaining 43 slots were available for sample probing and any eventually unused slot was filled with buffer. Hybridization was carried out at 54 °C for 60 min. The membrane was then washed twice at 62 °C for 10 min in 100 mL of 2× SSPE 0.5 %SDS buffer. Hybridized DNA was detected by streptavidin-peroxidase incubation (Spterptavidin-POD-conjugate, Roche) and enhanced chemiluminescence detection (ECL: enhanced chemo-luminescence detection kit; Amersham, Little Chalfont, UK), as described in the kit insert, followed by exposure to X-ray film (Hyperfilm ECL, Amersham, Little Chalfont, UK). The presence of a clearly visible black square was considered a positive hybridization reaction. All samples were evaluated in duplicate.

#### Stripping the membrane

For reuse, the membrane was stripped in 1% SDS solution at 80 °C (twice for 30–60 min) and rinsed in 20 mmol/L EDTA, pH 8.0, at room temperature. The membrane can be stripped and reused up to eight times without compromising the results.

### DNA sequencing

A 157 bp fragment of the *rpoB* gene was amplified using TR8 (5′-TGCACGTCGCGGACCTCCA-3′) and TR9 (5′-TCGCCGCGATCAAGGAGT-3′) primers. PCR was performed using 2.5 μL of 10 × buffer, 1.5 mmol/L MgCl_2_, 0.2 mmol/L of each dNTP, 0.4 μmol/L of each primer, 1 U/μL of Platinum Taq Polymerase (Invitrogen) and 5 μL (30 ng/μL) of template DNA in a 25 μL reaction volume under the following conditions: 15 min at 95 °C (initial denaturation), 35 cycles of 1 min at 94 °C (denaturation), 1 min at 58 °C (annealing), 1 min at 72 °C (extension), and one final step of 10 min at 72 °C (extension cycle) employing the PCR thermocycler ABI 9700 (Applied Biosystems). The amplified fragments were electrophoresed in 1% agarose gels and detected using ethidium bromide along with molecular-weight markers (100 bp DNA Ladder, Promega). The PCR products were purified using EXOSAP-IT (USB, USA) and bidirectionally sequenced on an ABI 3130xl automated sequencer (Applied Biosystems, Foster City, CA, USA), using BigDye Terminator version 1.1 Kits with the same primers used for the amplification. Analysis of electropherograms was done with the sequencing Analysis Mega 4 Software (Applied Biosystems). For each sample, PCR amplification and DNA sequencing were performed twice.

## Results and discussion

**Figure 1.**
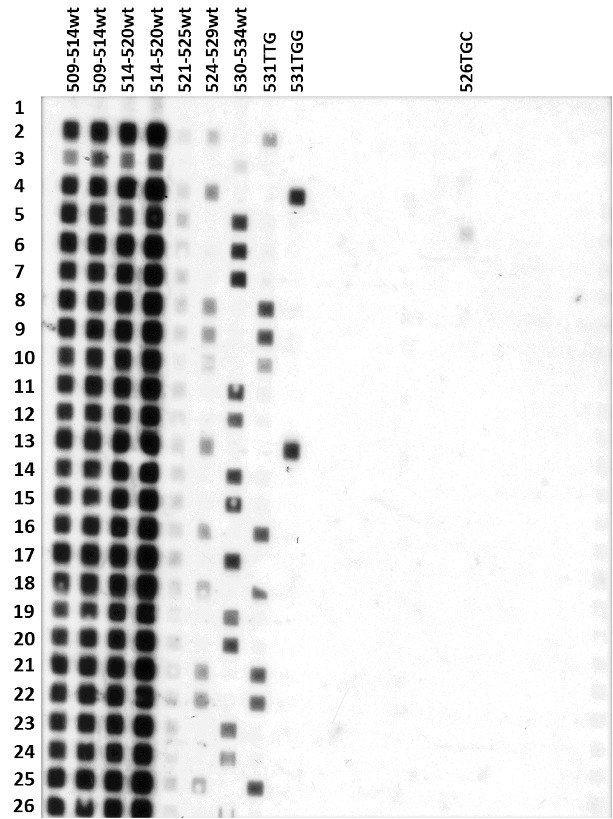
Typical result of the RIFO assay performed for the *rpoB* gene. Left to right: columns 1–7 contain blotted oligonucleotides corresponding to the wild-type (wt) sequence of the *rpoB* gene (duplicates of 509–514 wt and 514–520 wt, 521–525 wt, 524–529 wt and 530–534 wt) and columns 8–20 contain the mutant oligos loaded in the same order as described in Table 1. Top to bottom: rows 1–26 contain controls of rifampicin susceptible and resistant *M. tuberculosis* strains and patient samples (PCR products); row 1 is negative control (distilled H_2_O instead of DNA); rows 2–5 contain positive controls (531TTG, H37Rv(wt), Cp531TGG, Cp526TGC). Strains that lack hybridization to one of the five wild-type oligonucleotides are RIF resistant. Row 13 contains a sample that has a point mutation at position 531 of the *rpoB* gene (TGC → TGG). Strains in rows 8–10, 16, 18, 21, 22 and 25 bear the most common mutation: 531 TCG → TTG. Non-specific hybridization of the 524–529 wt probe.

A home-made test named RIFO for rifampicin oligonucleotide typing was applied to detect RIF resistant MTB strains, isolated from Moroccan patients. The RIFO method was applied on 500 clinical isolates of *M. tuberculosis* collected over a period of five years. Strains from the laboratory collection were used as known controls for the respective mutations, to improve the specificity and specificity of the test, both wild-type and mutant oligonucleotide probes were used. Initially, DST results showed that 154/500 (30.8%) isolates were phenotypically RIF resistant, whereas 346/500 (69.2%) isolates were RIF sensitive. When applying RIFO, accurate hybridization signals were obtained for all tested strains, except for the 524–529 wt (wild-type) and the 511 CCG mutant probe, which gave weak signals. A sample result of the RIFO assay is shown in Figure 1. This approach allows the identification of the point mutation in target codons of the *rpoB* gene; ten different missense mutations involving codons 511, 516, 522, 526 and 531 were identified in 140 strains (Table 2). Mutations at codon 531 were observed in 117 (83.6%) of the isolates, at codon 526 in 7(5%) isolates and at codon 516, in 13 (9.3%) isolates. The most common point mutations were Ser → Leu and Ser → Trp substitutions at codon 531, which were present, respectively, in 109 (77.9%) and 8 (5.7%) isolates, and Asp → Val point mutations in 9 (6.4%) isolates.

**Table 2.** RRDR mutations in the *rpoB* gene of 154 RIF^R^ strains of *M. tuberculosis* in Morocco identified by RIFO.

Codon	Nucleotide change	Amino-acid substitution	Frequency of mutations, *n* (%)
511	CTG → CCG	Leu → Pro	1
516	GAC → GTC	Asp → Val	9
516	GAC → TAC	Asp → Tyr	4
522	TCG → TTG	Ser → Leu	2
526	CAC → TGC	His → Cys	1
526	CAC → AAC	His → Asn	1
526	CAC → CTC	His → Leu	1
526	CAC → TAC	His → Tyr	4
531	TCG → TTG	Ser → Leu	109
531	TCG → TGG	Ser → Trp	8

To confirm the accuracy of the RIFO assay, a sub-sampling of 100 randomly selected strains was subjected to DNA sequencing of the RRDR region of the *rpoB* gene. For all strains that were revealed as genotypically resistant, sequencing analysis confirmed the point mutations identified by RIFO. Inversely, no mutation was detected by sequencing within RIF sensitive isolates.

A comparison of phenotypic resistance determined by conventional DST and genotypic resistance characterized by the presence of specific point mutations in the RRDR region of the *rpoB* gene is shown in Table 3. A total of 140 isolates were both phenotypically and genotypically resistant strains and 346 isolates were phenotypically sensitive, and RIFO confirmed the absence of any point mutation in the hot-spot region of the *rpoB* gene. However, discordance was obtained for 14 isolates that were phenotypically resistant strains but did not exhibit any point mutation in the hot-spot region associated with the resistance profile. Interestingly, all phenotypically sensitive strains had a wild-type sequence.

**Table 3.** Concordance between drug susceptibility and RIFO results.

Method and resistance status		DST results (conventional tests)		
	RIF resistant	Sensitive		Total
RIFO results	Mutant	140	0	140
	Wild type	14	346	360
Total		154	346	500

Based on these results, the specificity and sensitivity of the RIFO assay, as compared to the conventional DST, were calculated for the 500 isolates. The RIFO technique proved to have good sensitivity (90.9%) and high specificity (100%). The positive and negative predictive values were 100% and 96.1%, respectively.

Worldwide, the emergence and spread of drug resistance has been one of the greatest challenges facing the global efforts to control TB. The success of TB control programmes depends on the efficacy of TB diagnosis and early detection of TB resistance. Thus, there is a need for appropriate and inexpensive technologies to assess drug resistance for MTB, in order to optimize the use of limited resources in developing countries. Recent advances in molecular biology and new released methods have improved TB diagnosis and drug resistance detection.[33] However, these tools require investments in laboratory infrastructure, biosafety and staff specialization beyond the means of many resource-constrained settings where most patients live.

RIFO is a relatively easy test to perform in a laboratory with a medium level of technological capability. It is based on reverse line blot DNA hybridization for the detection of a panel of mutations in the core region of the *rpoB* gene.[29,32] Moreover, the platform described here is rather flexible and additional genes and specific probes can be incorporated for the detection of resistance to other drugs.[34,35]

Several assays were carried out to optimize the hybridization conditions and to achieve strong and neat hybridization signals. The most frequent mutations identified were Ser531Leu, Ser531Trp and Asp516Val. These findings are in agreement with previous studies on MTB isolates from Morocco and other settings.[12,18,23]

Compared to DNA sequencing, RIFO had high accuracy, as 100% of the point mutations observed by reverse line blot hybridization were confirmed by sequencing. Indeed, the concentration of probes and the stringency conditions of hybridization were optimized to give strong and highly specific hybridization.

The cornerstone of this study is the rapid detection of RIF^R^ strains by RIFO. Thus, among the 500 tested isolates, there were 140 strains with point mutations in the RRDR region of the *rpoB* gene conferring resistance to RIF with high positive and negative predictive values. The specificity and sensitivity of detection of RIFO are in concordance with the results obtained by Kourout et al. [18] using a dot blot hybridization approach and sequencing. The 14 false negative results corresponding to RIF^R^ strains that had no mutation could be explained by the fact that other mutations conferring resistance might occur in the *rpo*B gene elsewhere (or outside) the RRDR region, such as V146, or that changes have occurred in one or more genes whose products participate in antibiotic permeability or metabolism.[12,36–38]

An advantage of the approach is that the RRDR region of the *rpoB* gene is flanked by *M. tuberculosis* specific DNA sequences. Thus, it is possible to test for MTB and for RIF^R^ simultaneously by targeting a single amplicon generated using PCR technology.[39]

The overall cost of the test, including DNA extraction, PCR amplification and detection, is of particular interest and could be reduced because of possible reuse of the membrane up to eight times. Additionally, it allows the simultaneous analysis of 41 DNA samples and the oligonucleotide attachment is easy to scale up into a standardized format.

A particular advantage of molecular tests is their rapid turnaround time, especially in view of patient management and transmission of drug-resistant *M. tuberculosis*.[40,41] Notably, the RIFO assay has a turnaround time of less than 48 h, which makes it considerably faster than other conventional DST methods.[42,43] Another advantage of the RIFO assay reported here is that it follows the same format as spoligotyping, which is widely used in reference laboratories for simultaneous identification of MTB complex and strain-typing of MTB isolates.[44,45] Thus, with the same technology and using the same equipments and reagents, we can perform both RIFO for resistance genotyping and spoligotyping for epidemiological studies. An important issue that remains, however, is the affordability of molecular assays and the associated laboratory infrastructure needs in resource-constrained settings.[46]

The advantages of RIFO highlight it as an attractive tool for reference laboratories especially in high MDR-TB burden settings and resource-limited countries. Further ongoing developments of the assay include (1) the implementation of computer image analysis to reduce errors caused by subjective interpretation of the autoradiography, (2) the extension of the drug resistance coverage to other drugs, especially isoniazid and second-line drugs for rapid detection of MDR and XDR-TB.[45,46]

## Conclusions

The RIFO technique is an attempt to combine different targets (probes) in a single assay for prediction of RIF^R^. This rapid, simple, economical and highly sensitive and specific assay provides a practical alternative to sequencing for RIF^R^ genotyping to improve TB control management, especially in low-income countries.

## References

[cit0001] World Health Organization (WHO). Anti-Tuberculosis drug resistance in the world. Fourth Global Report. 2008. Geneve: World Health Organization. WHO/HTM/TB/2008.394.

[cit0002] Keshavjee S, Farmer PE (2012). Tuberculosis, drug resistance, and the history of modern medecine. N England J Med..

[cit0003] Gandhi NR, Nunn P, Dheda K, Schaaf HS, Zignol M, van Soolingen D, Jensen P, Bayona J (2010). Multidrug-resistant and extensively drug-resistant tuberculosis: a threat to global control of tuberculosis. Lancet..

[cit0004] Alcaide F, Coll P (2011). Advances in rapid diagnosis of tuberculosis disease and anti-tuberculous drug resistance. Enferm Infecc Microbiol Clin..

[cit0005] Johnson R, Streicher EM, Louw GE, Warren RM, van Helden PD, Victor TC (2006). Drug resistance in *Mycobacterium tuberculosis*. Curr Issues Mol Biol.

[cit0006] Sorlozano A, Soria I, Roman J, Huertas P, Soto MJ, Piedrola G, Gutierrez J (2009). Comparative evaluation of three culture methods for the isolation of mycobacteria from clinical samples. J Microbiol Biotechnol..

[cit0007] Tessema B, Beer J, Emmric F, Sack U, Rodloff AC. Analysis of gene mutations associated with isoniazid, rifampicin and ethambutol resistance among *Mycobacterium tuberculosis* isolates from Ethiopia. BMC Infect Dis. 2012;12:37. 10.1186/1471-2334-12-37 PMC3378438 22325147

[cit0008] Abe C (2003). Standardization of laboratory tests for tuberculosis and their proficiency testing. Kekkaku..

[cit0009] Simons SO, van Soolingen D (2011). Drug susceptibility testing for optimizing tuberculosis treatment. Curr Pharm Des..

[cit0010] Cole ST, Brosch R, Parkhill J, Garnier T, Churcher C, Harris D, Gordon SV, Eiglmeier K, Gas S, Barry CE, Tekaia F, Badcock K, Basham D, Brown D, Chillingworth T, Connor R, Davies R, Devlin K, Feltwell T, Gentles S, Hamlin N, Holroyd S, Hornsby T, Jagels K, Krogh A, McLean J, Moule S, Murphy L, Oliver K, Osborne J, Quail MA, Rajandream MA, Rogers J, Rutter S, Seeger K, Skelton J, Squares R, Squares S, Sulston JE, Taylor K, Whitehead S, Barrell BG (1998). Deciphering the biology of *Mycobacterium tuberculosis* from the complete genome sequence. Nature..

[cit0011] Musser JM (1995). Antimicrobial agent resistance in mycobacteria: molecular genetic insights. Clin Microbiol Rev..

[cit0012] Ramaswamy S, Musser JM (1998). Molecular genetic basis of antimicrobial agent resistance in *Mycobacterium tuberculosis*: update. Tuberc Lung Dis..

[cit0013] Lemus D, Martin A, Montoro E, Portaels F, Palomino JC (2004). Rapid alternative methods for detection of rifampicin resistance in *Mycobacterium tuberculosis*. J Antimicrob Chemother.

[cit0014] Miller LP, Crawford JT, Shinnick TM (1994). The *rpoB* gene of *Mycobacterium tuberculosis*. Antimicrob Agents Chemother..

[cit0015] Nisha A, Govindarajan S, Muthuraj M, Manupriya S, Usharani B, Kamatchyammal S, Saroja V (2012). Molecular characterization of *rpoB* gene encoding the RNA polymerase β subunit in rifampin-resistant *Mycobacterium tuberculosis* strains from south India. African J Biotechnol.

[cit0016] Cole ST, Telenti A (1995). Drug resistance in. Eur Respir J..

[cit0017] Sreevatsan S, Pan X, Stockbauer KE, Connell ND, Kreiswirth BN, Whittam TS, Musser JM (1997). Restricted structural gene polymorphism in the *Mycobacterium tuberculosis* complex indicates evolutionarily recent global dissemination. Proc Natl Acad..

[cit0018] Kourout M, Chaoui I, Sabouni R, Lahlou O, Mzibri M, Jordaan A, Victor TC, Akrim M, El Aouad R (2009). Molecular characterisation of rifampicin-resistant *Mycobacterium tuberculosis* strains from Morocco. Int J Tuberc Lung Dis..

[cit0019] Rattan A, Kalia A, Ahmad N (1998). Multidrug-resistant *Mycobacterium tuberculosis*: molecular perspectives. Emerg Infect Dis..

[cit0020] Jureen P, Werngren J, Hoffner SE (2004). Evaluation of the line probe assay (LiPA) for rapid detection of rifampicin resistance in *Mycobacterium tuberculosis*. Tuberculosis (Edinb).

[cit0021] Zhang SL, Shen JG, Xu PH, Li DX, Sun ZQ, Li L, Yang ZR, Sun Q (2007). A novel genotypic test for rapid detection of multidrug-resistant *Mycobacterium tuberculosis* isolates by a multiplex probe array. J Appl Microbiol.

[cit0022] Victor TC, Jordaan AM, van Rie A, van der Spuy GD, Richardson M, van Helden PD, Warren R (1999). Detection of mutations in drug resistance genes of *Mycobacterium tuberculosis* by a dot-blot hybridization strategy. Tuberc Lung Dis..

[cit0023] Van Rie A, Warren R, Mshanga I, Jordaan AM, van derSpuy GD, Richardson M, Simpson J, Gie RP, Enarson DA, Beyers N, van Helden PD, Victor TC (2001). Analysis for a limited number of gene codons can predict drug resistance of *Mycobacterium tuberculosis* in a high-incidence community. J Clin Microbiol..

[cit0024] Victor TC, van Helden PD, Warren R (2002). Prediction of drug resistance in *Mycobacterium tuberculosis*: molecular mechanisms, tools, and applications. IUBMB Life.

[cit0025] Weyer K, Mirzayev F, Migliori G, Gemert W, D’Ambrosio L, Zignol M, Floyd K, Centis R, Cirillo D, Tortoli E, Gilpin C, Iragena J, Falzon D, Raviglione M (2013). Rapid molecular TB diagnosis: evidence, policy-making and global implementation of Xpert(R) MTB/RIF. Eur Respir J..

[cit0026] Piatek AS,, Tyagi S,, Pol AC,, Telenti A,, Miller LP,, Kramer FR,, Alland D (1998). Molecular beacon sequence analysis for detecting drug resistance in *Mycobacterium tuberculosis*. Nat Biotechnol.

[cit0027] El-Hajj HH, Marras SA,, Tyagi S,, Kramer FR,, Alland D (2001). Detection of rifampin resistance in *Mycobacterium tuberculosis* in a single tube with molecular beacons. J Clin Microbiol.

[cit0028] Boehme CC, Nabeta P,, Hillemann D,, Nicol MP,, Shenai S,, Krapp F,, Allen J,, Tahirli R,, Blakemore R,, Rustomjee R,, Milovic A,, Jones M,, O’Brien SM,, Persing DH, Ruesch-Gerdes S,, Gotuzzo E,, Rodrigues C,, Alland D,, Perkins MD (2010). Rapid molecular detection of tuberculosis and rifampin resistance. N England J Med..

[cit0029] Morcillo N, Zumarraga M, Alito A, Dolmann A, Schouls L, Cataldi A, Kremer K, van Soolingen D (2002). A low cost, home-made, reverse-line blot hybridisation assay for rapid detection of rifampicin resistance in *Mycobacterium tuberculosis*. Int J Tuberc Lung Dis..

[cit0030] Canetti G, Froman S, Grosset J, Hauduroy P, Langerova M, Mahler HT, Meissner G, Mitchison DA, Sula L (1963). Mycobacteria: laboratory methods for testing drug sensitivity and resistance. Bull World Health Organ..

[cit0031] Canetti G, Fox W, Khomenko A, Mahler HT, Menon NK, Mitchison DA, Rist N, Smelev NA (1969). Advances in techniques of testing mycobacterial drug sensitivity, and the use of sensitivity tests in tuberculosis control programmes. Bull World Health Organ..

[cit0032] Senna SG, Gomes HM, Ribeiro MO, Kristki AL, Rossetti ML, Suffys PN (2006). In house reverse line hybridization assay for rapid detection of susceptibility to rifampicin in isolates of *Mycobacterium tuberculosis*. J Microbiol Methods..

[cit0033] Dorman SE (2010). New diagnostic tests for tuberculosis: bench, bedside, and beyond. Clin Infect Dis..

[cit0034] Mokrousov I, Bhanu NV, Suffys PN, Kadival GV, Yap SF, Cho SN, Jordaan AM, Narvskaya O, Singh UB, Gomes HM, Lee H, Kulkarni SP, Lim KC, Khan BK, van Soolingen D, Victor TC, Schouls LM (2004). Multicenter evaluation of reverse line blot assay for detection of drug resistance in *Mycobacterium tuberculosis* clinical isolates. J Microbiol Methods..

[cit0035] Ajbani K, Shetty A, Mehta A, Rodrigues C (2011). Rapid diagnosis of extensively Drug-resistant tuberculosis by use of reverse line blot hybridization assay. J Clin Microbiol..

[cit0036] Heep M, Brandstatter B, Rieger U, Lehn N, Richter E, Rüsch-Gerdes S, Niemann S (2001). Frequency of *rpoB* mutations inside and outside the cluster I region in rifampin-resistant clinical *Mycobacterium tuberculosis* isolates. J Clin Microbiol..

[cit0037] Kapur V, Li LL, Iordanescu S, Hamrick MR, Wanger A, Kreiswirth BN, Musser JM (1994). Characterization by automated DNA sequencing of mutations in the gene (*rpoB*) encoding the RNA polymerase beta subunit in rifampin-resistant *Mycobacterium tuberculosis* strains from New York City and Texas. J Clin Microbiol..

[cit0038] Patra SK, Jain A, Sherwal BL, Khanna A (2010). Rapid Detection of Mutation in RRDR of *rpoB* gene for Rifampicin Resistance in MDR-Pulmonary Tuberculosis by DNA Sequencing. Indian J Clin Biochem..

[cit0039] Lawn SD, Nicol MP (2011). Xpert® MTB/RIF assay: development, evaluation and implementation of a new rapid molecular diagnostic for tuberculosis and rifampicin resistance. Future Microbiol..

[cit0040] Ling DI, Zwerling AA, Pai M (2008). GenoType MTBDR assays for the diagnosis of multidrug-resistant tuberculosis: a meta-analysis. Eur Respir J..

[cit0041] Xu HB,, Jiang RH,, Sha W,, Li L,, Xiao HP (2010). PCR-single–strand conformational polymorphism method for rapid detection of rifampin-resistant *Mycobacterium tuberculosis* : systematic review and meta-analysis. J Clin Microbiol.

[cit0042] Kremer K, van Zetten, van Embden J, Shouls L, van Soolingen D (2003). ‘RIFO ASSAY’: a PCR reverse line blot hybridization method to detect rifampin resistance.

[cit0043] Suresh N, Singh UB, Arora J, Pande JN, Seth P, Samantaray JC (2006). Rapid detection of rifampicin-resistant *Mycobacterium tuberculosis* by in-house, reverse line blot assay. Diagn Microbiol Infect Dis..

[cit0044] Kamerbeek J, Schouls L, Kolk A, van Agterveld M, van Soolingen D, Kuijper S, Bunschoten A, Molhuizen H, Shaw R, Goyal M, van Embden J (1997). Simultaneous detection and strain differentiation of *Mycobacterium tuberculosis* for diagnosis and epidemiology. J Clin Microbiol.

[cit0045] Neuta IH, Varela A, Martin A, von Groll A, Jureen P, López B, Imperiale B, Sķenders G, Ritacco V, Hoffner S, Morcillo N, Palomino JC, Del Portillo P (2010). Rifampin-isoniazid oligonucleotide typing: an alternative format for rapid detection of multidrug-resistant *Mycobacterium tuberculosis*. J Clin Microbiol.

[cit0046] Brossier F, Veziris N, Truffot-Pernot C, Jarlier V, Sougakoff W (2006). Performance of the genotype MTBDR line probe assay for detection of resistance to rifampin and isoniazid in strains of *Mycobacterium tuberculosis* with low- and high-level resistance. J Clin Microbiol.

